# High Myoinositol on Proton MR Spectroscopy Could Be a Potential Signature of Papillary Tumors of the Pineal Region—Case Report of Two Patients

**DOI:** 10.3390/brainsci12060802

**Published:** 2022-06-19

**Authors:** Albert Pons-Escoda, Juan Jose Sánchez Fernández, Àlex de Vilalta, Noemí Vidal, Carles Majós

**Affiliations:** 1 Institut de Diagnóstic per la Imatge (IDI), Department of Radiology, Hospital de Bellvitge, 08908 L’Hospitalet de Llobregat, Barcelona, Spain; juanjo.sanchez.idi@gencat.cat (J.J.S.F.); carles.majos.idi@gencat.cat (C.M.); 2 Neuro-Oncology Unit, Institut d’Investigació Biomèdica de Bellvitge (IDIBELL), 08908 L’Hospitalet de Llobregat, Barcelona, Spain; nvidal@bellvitgehospital.cat; 3 Deparment of Neurosurgery, Hospital de Bellvitge, 08908 L’Hospitalet de Llobregat, Barcelona, Spain; avilalta@bellvitgehospital.cat; 4 Deparment of Pathology, Hospital de Bellvitge, 08908 L’Hospitalet de Llobregat, Barcelona, Spain

**Keywords:** pineal neoplasms, differential diagnosis, NMR spectroscopy, myoinositol

## Abstract

Papillary tumor of the pineal region (PTPR) is an uncommon entity in which a presurgical suspicion may be crucial for patient management. Maximal safe neurosurgical resection is of choice when PTPR is suspected, whereas non-surgical approaches can be considered in other tumors of the pineal region, such as pineocytoma or concrete subtypes of germ-cell tumors. In general terms, imaging features of tumors of the pineal region have been reported to be unspecific. Nevertheless, in this report, we describe two pathology-confirmed PTPRs in which presurgical proton MR spectroscopy demonstrated extremely high myoinositol, a pattern which drastically differs from that of other pineal tumors. We hypothesize that this high myoinositol may be related to PTPR’s known ependymal component, and that it could be used as a specific non-invasive diagnostic signature.

## 1. Introduction

Various histological types of tumors arise in the pineal region. The most common are parenchymal pineal tumors (PPT) and germ-cell tumors (GCT) [1]. Papillary tumor of the pineal region (PTPR) is a rare tumor entity first described in 2003 [2], and later codified in the 2007 WHO classification of central nervous system tumors [3]. In the 2021 WHO classification, it appears as one of five subtypes of PPT, along with pineocytoma, PPT of intermediate differentiation, pineoblastoma, and desmoplastic myxoid tumor of the pineal region SMARCB-1-mutant [4].

Presurgical identification of this WHO grade II-III tumor can be very relevant for patient management. Though conservative follow-up strategies are accepted in other PPTs, such as WHO grade I pineocytoma, and surgical resection may be pointless in chemoradiotherapy-sensitive germ-cell tumors (GCT), gross total resection is considered the treatment of choice for PTPR, and has been related to improved outcomes [5]. Unfortunately, the radiological appearance of pineal gland tumors is often unspecific, and a confident presurgical diagnosis may not be reached based on morphological imaging alone [4,5,6,7].

There is little literature about PTPR advanced imaging features, mainly based on case reports [8].

Here, we report two pathology-confirmed cases of PTPR in which proton MR spectroscopy (1H-MRS) was performed. A very high myoinositol peak predominated in the spectra of both cases; a finding which may be related to PTPR’s known ependymal component, and which provides a signature that could be distinctive of PTPR.

## 2. Case Report

Included patients gave written informed consent in using their data for educational and research purposes.

### 2.1. Case 1

**Figure 1:**
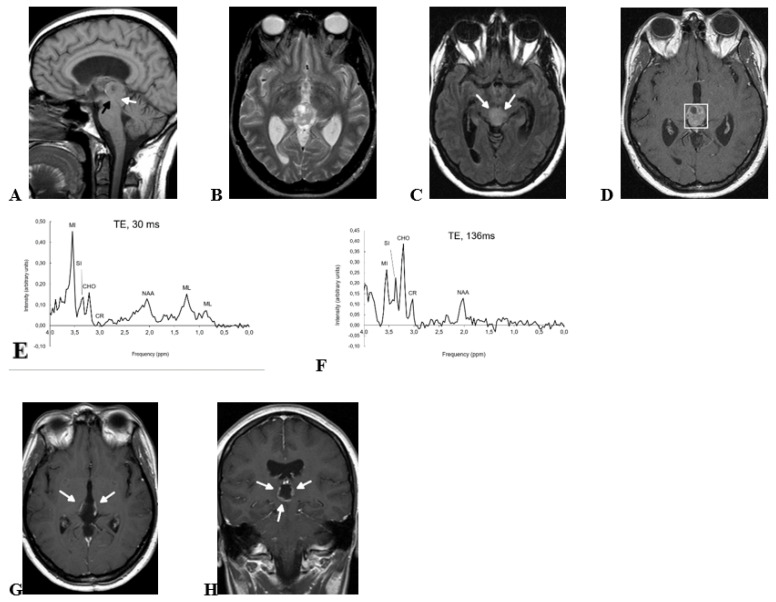
Preoperative MRI (**A**–**D**), preoperative 1H-MRS (**E**,**F**), and postoperative MRI (**G**,**H**) of a 30-yr-old woman with pathology-confirmed PTPR. (**A**) Sagital T1-wi show an inhomogeneous pineal region mass with small areas of cystic degeneration. Sylvian aqueduct compression secondary to the mass (black arrow) and midbrain tegmentum infiltration (white arrow, see also Figure 1C) are shown in the image. (**B**) Axial T2-wi show that the mass signal is heterogeneous with multiple small cystic areas. Tumor margins are well defined at this level, and slight mass-effect is produced to both thalami. (**C**) Axial FLAIR-wi at a lower level than B shows midbrain infiltration (white arrows). (**D**) Contrast-enhanced T1-wi confirms a well-defined mass at this level with small cystic areas and non-homogeneous enhancement. Voxel for spectroscopy is shown. (**E**) Short TE 1H-MRS (TE, 30 ms) shows very high myoinositol signal at 3.55 ppm. (**F**) Long TE 1H-MRS (TE, 136 ms) shows that the intensity at 3.55 ppm is reduced at this echo time due to short T2 relaxation time of myoinositol. MI, myoinositol; SI, scyllo-inositol; CHO, choline; CR, creatine; NAA, N-acetyl-aspartate; ML, mobile lipids. (**G**,**H**) Postoperative axial and coronal contrast-enhanced T1-wi show annular contrast enhancement, suggesting partial surgical resection (arrows). Adjuvant treatment with radiotherapy was administered, and contrast enhancement disappeared in follow-up (not shown).

A 30-year-old woman was admitted to our emergency room with sudden-onset headache, nausea, vomiting, and diplopia. CT and MRI exams were performed, and showed a 23 mm heterogeneous mass centered in the pineal region with small cystic areas (Figure 1). The mass was of intermediate intensity signal on T1-wi and T2-wi, and hyperintense on FLAIR-wi. Moderate contrast enhancement was found after contrast administration on T1-wi. An inhomogeneous diffusion restriction was observed in diffusion-wi. Midbrain infiltration was seen on axial FLAIR-wi and sagittal T1-wi (white arrows in Figure 1A,C). Mass-effect related to the lesion produced displacement of the postero-medial aspect of both thalami, deformity of the posterior III ventricle, and Sylvian aqueduct stenosis (black arrow in Figure 1A). Secondary obstructive supratentorial hydrocephalus was found related to mass-effect. Single voxel 1H-MRS was performed at short and long TE. Parameters for spectroscopy were the following: PRESS; TE, both 30 and 136 ms; TR, 2.000 ms; VOI, 2 × 2 × 2 mm; averages, 128. High choline/creatine and choline/NAA ratios were found both at short and long TE, jointly with small amounts of mobile lipids at short TE centered at 0.9 and 1.3 ppm. However, the most prominent finding was a high myoinositol peak at short TE centered at 3.55 ppm, much higher than any other resonance in the spectrum (myoinositol/choline ratio = 2.87, myoinositol/creatine = 25.17). The resonance signal at 3.55 ppm was significantly reduced at long TE, supporting myoinositol as the main origin of the peak. The tumor markers, alpha-fetoprotein and beta-HCG, were measured in blood and were found in normal ranges. Based on these findings, resective surgery was performed, and histopathology evaluation reported findings compatible with PTPR. Post-operative MRI demonstrated ring-like enhancement in the pineal area, suggesting laminar tumor remnant (arrows in Figure 1G,H). Post-operative patient performance status was satisfactory (Karnofsky Performance Status = 90), with sporadic headaches being the patient’s only complaint after surgery. Adjuvant treatment with radiotherapy was indicated, and 50 Gy were administered to the enhancing area in 25 sessions. Enhancement in CE-T1-wi disappeared in successive follow-up, and the patient remained asymptomatic except for sporadic headaches until she was lost at follow-up after 10 years.

### 2.2. Case 2

**Figure 2:**
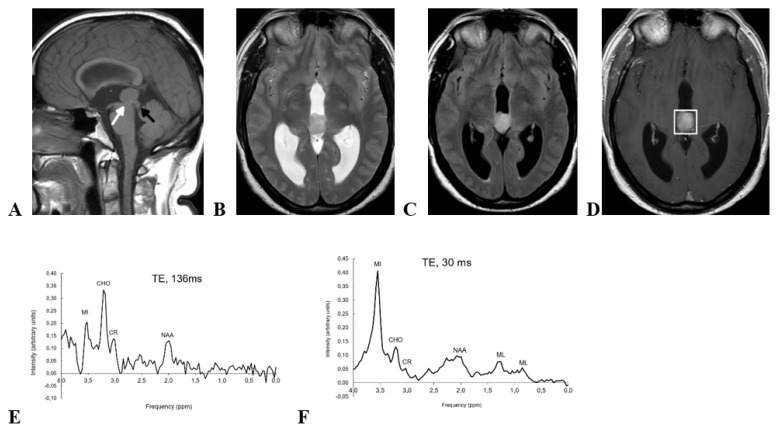
Preoperative MRI and 1H-MRS of a 52-yr-old man with pathology-confirmed PTPR. (**A**) Sagital T1-wi shows a homogeneous solid mass centered in the pineal region of intermediate intensity signal on this sequence. Note: midbrain tegmentum displacement (black arrow) and Sylvian aqueduct stenosis (white arrow). (**B**) Axial T2-wi and (**C**) Axial FLAIR-wi show that the mass is slightly hyperintense in both sequences. (**D**) Axial CET1-wi shows well defined margins of the mass are well-defined, and slightly inhomogeneous contrast enhancements. Voxel position for spectroscopy is shown. (**E**) Short TE 1H-MRS (TE, 30 ms) shows a huge myoinositol peak at 3.55 ppm. (**F**) Long TE 1H-MRS (TE, 136 ms) with signal loss at 3.55 ppm that confirms preponderant myoinositol contribution. MI, myoinositol; CHO, choline; CR, creatine; NAA, N-acetyl-aspartate; ML, mobile lipids.

A 52-year-old man presented with a 3-week history of headache and paresthesia in lower extremities, along with 2-months history of instability, nausea, and gait disturbance. MRI showed a 21 mm solid mass centered in the pineal region and growing into the III ventricle (Figure 2). The mass was slightly hyperintense on T2-wi and FLAIR-wi, and showed slightly inhomogeneous enhancement on contrast-enhanced T1-wi. An inhomogeneous diffusion restriction was observed in diffusion-wi. Midbrain tegmentum was displaced by the mass (black arrow in Figure 2A) without imaging signs of infiltration. The Sylvian aqueduct was compressed (white arrow in Figure 2A) with consequent supratentorial hydrocephalus. Single voxel 1H-MRS was performed with the same parameters used in case 1 (PRESS; TE, 30 and 136 ms; TR, 2.000 ms; VOI, 2 × 2 × 2 mm; averages, 128). The findings on 1H-MRS were similar to case 1: high choline/creatine and choline/NAA ratios at short and long echo-times, small amounts of mobile lipids centered at 0.9 and 1.3 ppm only at short TE, and very high myoinositol at short TE as the most prominent finding (myoinositol/choline ratio = 3.10, myoinositol/creatine = 7.66). Endoscopic ventriculostomy with simultaneous biopsy was performed, and histopathology revealed a PTPR. Then, resective surgery was indicated and further performed a second time. Histopathology evaluation confirmed a PTPR. Post-operative MRI (not shown) showed a complete resection with no tumor remnant depictable. Parinaud syndrome and IV cranial nerve impairment was detected in early postoperative neuro-ophthalmology exam, and partially improved in follow-up. Close follow-up without further adjuvant treatment was decided in the multidisciplinary neurooncology unit. The patient showed progressive improvement of neuro-ophthalmology symptoms and good performance status (Karnofsky Performance Status = 100) after 6-months follow-up.

## 3. Discussion

Less than 1% of all intracranial tumors are in the pineal region, with PPT and GCT being the most frequent entities [1]. PTPR is a very uncommon tumor of the pineal region, and it is one of five PPT subtypes according to the 2021 WHO classification of central nervous system tumors [4]. Although the prevalence of PTPR is low, recognition of this tumor type prior to surgery can be very relevant for patient management. Conservative follow-up can be considered in lower grade PPTs, such as pineocytoma. On the other hand, surgical resection may be pointless in chemoradiotherapy-sensitive GCT, in which treatment with chemoradiotherapy after biopsy may be a better choice. On the contrary, PTPRs are tumors of intermediate malignancy according to the WHO classification, and maximal safe surgical resection should be considered the treatment of choice [4,5]. Unfortunately, the radiological appearance of pineal tumors is described to be relatively unspecific with few imaging pearls: PPTs displace normal pineal calcifications towards the periphery; pineoblastomas show marked restricted diffusion with low ADC values; pineal teratomas display fat signal; pineal cysts and arachnoid cysts show cerebrospinal fluid signal; and pineal lesions in patients with known malignancy should raise suspicion of metastatic involvement. PTPRs usually appear as well-defined solid masses with predominantly intermediate signal on T2-wi, areas of cystic degeneration, and slightly heterogeneous contrast enhancement. This radiological pattern can be very similar to those of other tumors of the pineal region, and no conventional imaging clues are described to allow a confident presurgical suspicion of PTPR [4,5,6,7]. This is in line with our experience based on the two reported cases, which presented unspecific features on conventional MRI. Regarding advanced MRI, Vaghela et al. reported high cerebral blood volume, little diffusion restriction, and, in line with our current reports, a presence of myoinositol peak as important imaging findings for this entity [8].

1H-MRS is a non-invasive MR technique which provides biochemical information on the tissues. It concretely provides graphics that enable the evaluation of the relative amounts of several metabolites on a concrete region of interest. Indeed, 1H-MRS is known to be useful in providing additional information for brain tumors’ presurgical diagnosis [8,9]. In this respect, specific metabolic signatures have been reported to be of great aid in some particular tumor types, e.g., meningioma and solitary fibrous tumors (previously known as hemangiopericytomas) are dural tumors that are not reliably differentiable based on morphological imaging. Nevertheless, meningioma is known to characteristically show high amounts of alanine and glutamine-glutamate, whereas the solitary fibrous tumor 1H-MRS pattern is characterized by high myoinositol. These 1H-MRS findings often allow these two entities presurgical differentiation, which may be very relevant for patient management [10]. With this exemplifying reference in mind, we believe the spectroscopic pattern of PTPR may provide similar presurgical diagnostic aid when facing a tumor of the pineal region. Indeed, both PTPR reported in this manuscript showed very high myoinositol as the most prominent peak in the spectrum, and this finding may constitute a biomarker to suggest a PTPR when a tumor is found in the pineal region.

**Figure 3:**
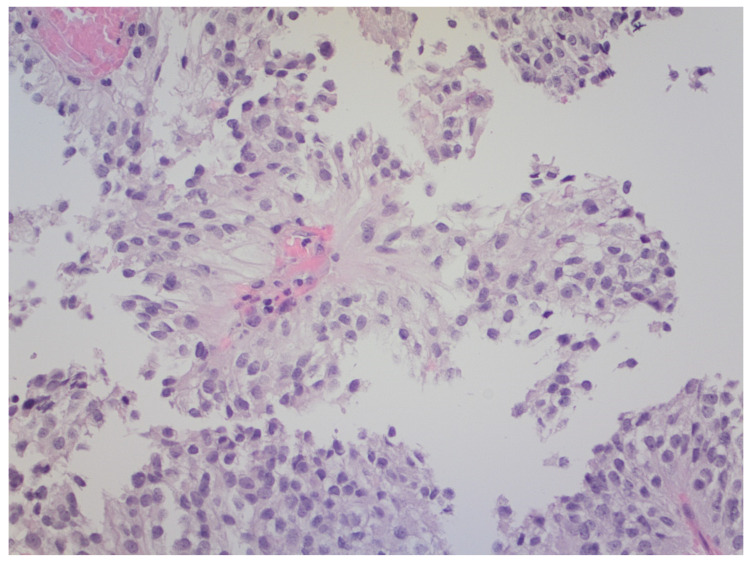
Hematoxylin-eosin staining ×20. PTPR with prominent papillary architecture. The vessels are covered by layers of large, eosinophilic columnar cells. The nuclei are round to oval, with stippled chromatin.

PTPR is thought to originate from a distinct ependymal cell of the subcommissural organ [2]. On histopathology, PTPR is characterized by an epithelial-like, loose papillary growth pattern similar to ependymomas [11] (Figure 3). In fact, PTPR shares some features with ependymomas on immunohistochemistry [11], and on the basis of our two reported cases, they could also share metabolic features depicted by 1H-MRS. Our two cases showed very prominent peaks of myoinositol at 3.55 ppm on short TE spectroscopy, concretely more than twice than any other resonance in the spectrum. Myoinositol is a sugar that plays a role in osmotic pressure and cell volume regulation. It is increased in gliomas, and it may be considered a glial cell biomarker. Astrocytomas show different degrees of myoinositol, and this finding has been useful in astrocytoma grading, genetic profiling, and prognostic stratification [12,13,14,15,16], as well as indiscriminating astrocytoma from pseudotumoral lesions [17] and metastases [18]. In the case of ependymal tumors, myoinositol is not only very high, but also constitutes the main resonance on the spectrum being much higher than in other gliomas. In this respect, Mora P et al. reported that high myoinositol in a posterior fossa tumor can be considered a metabolic signature of ependymoma [19]. In our two cases, the levels of myoinositol were in the range of ependymomas, extremely different from what has been reported in other pineal tumors. On 1H-MRS, GCT and the other PPTs have been reported to show high choline and low NAA. Harris LM et al. reported taurine as a characteristic signature of GCT and pineoblastomas, and high lipids and macromolecules a signature of GCT. Small amounts of myoinositol have also been reported in pineoblastomas [20,21]. However, myoinositol levels in our two cases were much higher, yielding a spectral pattern superimposable on the ependymoma’s. Therefore, we hypothesize that the high amounts of myoinositol found in PTPR may be related to the known ependymal origin of this tumor. To summarize, in accordance with all exposed facts: finding high amounts of myoinositol in a pineal region tumor could suggest an ependymal component of the tumor, and, consequently, a presurgical suspicion of PTPR.

The current manuscript is a case report based on two single cases, with its inherent limitations. Nevertheless, the authors believe that its importance lays in the reported spectroscopic pattern that may help neuroradiologists in every-day practice when facing a pineal tumor, and also in promoting necessary further research studies in this regard.

## 4. Conclusions

PTPR 1H-MRS pattern is characterized by very high myoinositol as the most prominent resonance in the spectrum. This finding could be relevant for the diagnosis and management of pineal tumors in two ways: (1) short TE 1H-MRS must be obtained when a pineal tumor is found on imaging; and (2) if high myoinositol is confirmed, PTPR should be presurgically diagnosed, and prompt gross tumor resection should be considered.

## Abbreviations

1H-MRS: proton magnetic resonance spectroscopy; GCT, germ cell tumors; NAA, N-acetyl aspartate; PPT, parenchymal pineal tumors; PRESS, point-resolved spectroscopy; PTPR, papillary tumor of the pineal region; TE, echo time; TR, repetition time; VOI, volume of interest.

## References

[1] Hirato J., Nakazato Y. (2001). Pathology of pineal region tumors. J. Neurooncol..

[2] Jouvet A., Fauchon F., Liberski P., Saint-Pierre G., Didier-Bazes M., Heitzmann A., Delisle M.B., Biassette H.A., Vincent S., Mikol J. (2003). Papillary Tumor of the Pineal Region. Am. J. Surg. Pathol..

[3] Louis D.N., Ohgaki H., Wiestler O.D., Cavenee W.K., Burger P.C., Jouvet A., Scheithauer B.W., Kleihues P. (2007). The 2007 WHO Classification of Tumours of the Central Nervous System. Acta Neuropathol..

[4] WHO Classification of Tumours Editorial Board (2021). World Health Organization Classification of Tumours of the Central Nervous System.

[5] Yamaki V.N., Solla D.J.F., Ribeiro R.R., da Silva S.A., Teixeira M.J., Figueiredo E.G. (2019). Papillary Tumor of the Pineal Region: Systematic Review and Analysis of Prognostic Factors. Clin. Neurosurg..

[6] Mathkour M., Hanna J., Ibrahim N., Scullen T., Kilgore M.D., Werner C., Cormier I., Spencer P., Keen J.R., Bui C.J. (2021). Papillary tumor of the pineal region in pediatric populations: An additional case and systematic review of a rare tumor entity. Clin. Neurol. Neurosurg..

[7] Fang A.S., Meyers S.P. (2013). Magnetic resonance imaging of pineal region tumours. Insights Imaging.

[8] Vaghela V., Radhakrishnan N., Radhakrishnan V.V., Menon G., Kesavadas C., Thomas B. (2010). Advanced magnetic resonance imaging with histopathological correlation in papillary tumor of pineal region: Report of a case and review of literature. Neurol. India.

[9] Tate A.R., Underwood J., Acosta D.M., Julià-Sapé M., Majós C., Moreno-Torres A., Howe F.A., van der Graaf M., Lefournier V., Murphy M.M. (2006). Development of a decision support system for diagnosis and grading of brain tumours using in vivo magnetic resonance single voxel spectra. NMR Biomed..

[10] Barba I., Moreno A., Martínez-Pérez I., Tate A.R., Cabañas M.E., Baquero M., Capdevila A., Arús C. (2001). Magnetic resonance spectroscopy of brain hemangiopericytomas: High myoinositol concentrations and discrimination from meningiomas. J. Neurosurg..

[11] Heim S., Sill M., Jones D.T.W., Vasiljevic A., Jouvet A., Fèvre-Montange M., Wesseling P., Beschorner R., Mittelbronn M., Kohlhof P. (2016). Papillary tumor of the pineal region: A distinct molecular entity. Brain Pathol..

[12] Castillo M., Smith J.K., Kwock L. (2000). Correlation of myo-inositol levels and grading of cerebral astrocytomas. AJNR Am. J. Neuroradiol..

[13] Bulik M., Jancalek R., Vanicek J., Skoch A., Mechl M. (2013). Potential of MR spectroscopy for assessment of glioma grading. Clin. Neurol. Neurosurg..

[14] Stadlbauer A., Gruber S., Nimsky C., Fahlbusch R., Hammen T., Buslei R., Tomandl B., Moser E., Ganslandt O. (2006). Preoperative grading of gliomas by using metabolite quantification with high-spatial-resolution proton MR spectroscopic imaging. Radiology.

[15] Nakae S., Murayama K., Sasaki H., Kumon M., Nishiyama Y., Ohba S., Adachi K., Nagahisa S., Hayashi T., Inamasu J. (2017). Prediction of genetic subgroups in adult supra tentorial gliomas by pre- and intraoperative parameters. J. Neurooncol..

[16] Kumon M., Nakae S., Murayama K., Kato T., Ohba S., Inamasu J., Yamada S., Abe M., Sasaki H., Ohno Y. (2021). Myoinositol to Total Choline Ratio in Glioblastomas as a Potential Prognostic Factor in Preoperative Magnetic Resonance Spectroscopy. Neurol. Med. Chir. (Tokyo).

[17] Majós C., Aguilera C., Alonso J., Julià-Sapé M., Castañer S., Sanchez J.J., Samitier A., León A., Rovira A., Arús C. (2009). Proton MR Spectroscopy Improves Discrimination between Tumor and Pseudotumoral Lesion in Solid Brain Masses. Am. J. Neuroradiol..

[18] Fan G., Sun B., Wu Z., Guo Q., Guo Y. (2004). In vivo single-voxel proton MR spectroscopy in the differentiation of high-grade gliomas and solitary metastases. Clin. Radiol..

[19] Mora P., Pons A., Cos M., Camins A., Muntané A., Aguilera C., Arús C., Majós C. (2019). Magnetic resonance spectroscopy in posterior fossa tumours: The tumour spectroscopic signature may improve discrimination in adults among haemangioblastoma, ependymal tumours, medulloblastoma, and metastasis. Eur. Radiol..

[20] Tamrazi B., Nelson M., Blüml S. (2017). Pineal Region Masses in Pediatric Patients. Neuroimaging Clin. N Am..

[21] Harris L.M., Davies N., Wilson S., MacPherson L., Natarajan K., English M.W., Brundler M.-A., Arvanitis T., Grundy R.G., Peet A.C. (2011). Short echo time single voxel 1H magnetic resonance spectroscopy in the diagnosis and characterisation of pineal tumours in children. Pediatr. Blood Cancer.

